# Penicillin Allergy and Perioperative Anaphylaxis

**DOI:** 10.3389/falgy.2022.903161

**Published:** 2022-06-09

**Authors:** Allison Ramsey

**Affiliations:** ^1^Rochester Regional Health, Rochester, NY, United States; ^2^Clinical Assistant Professor of Medicine, University of Rochester School of Medicine and Dentistry, Rochester, NY, United States

**Keywords:** penicillin allergy, perioperative anaphylaxis, penicillin skin testing, pregnancy, preoperative, cephalosporin—drug

## Abstract

Penicillin allergy is the most commonly reported drug allergy, while perioperative anaphylaxis is overall rare. This review covers the epidemiology of both penicillin allergy and perioperative anaphylaxis both separately and taken together. Considerations regarding anaphylaxis to penicillin during pregnancy are also discussed, since penicillin is the drug of choice for Group B *Streptococcus* prophylaxis. The minimal cross reactivity between penicillins and cephalosporins is addressed, since the vast majority of patients with a penicillin allergy label can receive perioperative cephalosporins. The management of the patient who has experienced perioperative anaphylaxis, including the importance of allergy referral is covered. Approaches to pre-operative penicillin allergy evaluations and opportunities for education are highlighted.

## Introduction

Penicillin allergy is commonly reported, occurring in about 10% of the population (1), while perioperative anaphylaxis is rare, with severe episodes estimated to occur in 1 in 10,000 surgical procedures (2), though this latter figure may be an underestimate (3). Since penicillin allergy is common, and surgical procedures in the US exceed 50 million per year (4), it is worthwhile to consider penicillin allergy in the context of perioperative anaphylaxis and vice versa. This review covers a brief description of the epidemiology of both perioperative anaphylaxis and penicillin allergy, the epidemiology of perioperative anaphylaxis attributable to penicillin-based antibiotics, and the approach to the patient with perioperative anaphylaxis due to a penicillin-based antibiotic, including in pregnancy. It also includes a discussion surrounding the use of perioperative cephalosporins in the setting of penicillin allergy, the evidence surrounding pre-operative penicillin allergy evaluations, and highlights directions for future study.

## Epidemiology of Penicillin Allergy and Perioperative Anaphylaxis

The adverse outcomes associated with the penicillin allergy label are well-documented, and occur at both individual and public health levels (Table 1). For the individual, a penicillin allergy label is associated with lower cure rates, increased recurrence rates, increased risks for adverse effects from second line antibiotics, and increased mortality (1, 5, 6). On a public health level, a penicillin allergy is associated with higher rates of *Clostridiodes difficile* and methicillin-resistant *Staphylococcus aureus*, along with longer and more costly hospital stays (5, 7). Most germane to this review, the penicillin allergy label has been associated with an increased risk of surgical site infections (8).

**Table 1:** Penicillin allergy and perioperative anaphylaxis characteristics.

	**Penicillin allergy**	**Perioperative anaphylaxis**
Incidence	10% of US general population	0.015 % of US surgical procedures
Sex predominance	Female > Male	Female > Male
Considerations	Risks of label: decreased cure rates, increased costs, increased surgical site infection, mortality	Most common causes: β-lactam antibiotics (US and UK), neuromuscular blocking agents (Europe)
**Both**	**Overall rare, most common in US/UK**, **allergy/immunology referral indicated**	

Perioperative anaphylaxis is an acute, systemic, life threatening reaction that occurs during the operative period. It is most common in females, and can be more severe due to many characteristics unique to the perioperative setting, including sedated patients who are draped for surgery (9). Both IgE and non-IgE mediated mechanisms have been recognized (2). The most common agents implicated in perioperative anaphylaxis are antibiotics, neuromuscular blocking agents (NMBA), sugammadex (an NMBA reversal agent), chlorhexidine, and latex (2). The most recent US data of perioperative anaphylaxis was published by Gonzalez-Estrada and colleagues, using the National Inpatient Sample from 2005–2014 (10, 11). The incidence of perioperative anaphylaxis was 1 in 6,531 procedures, with 5% of cases near fatal and 2% fatal (10, 11). Study methodology prevented evaluation for casual agents (10). A multicenter study from the UK and Ireland looked at the epidemiology of perioperative anaphylaxis and found that the reported incidence of perioperative anaphylaxis of 1:1,300–1:13,000 may be underestimated, and found an incidence of 1:353 in their analysis (12). A 5 year series conducted in Spain identified 48 cases of perioperative anaphylaxis, and also demonstrated that antibiotics and NMBAs were the most common cause, based on subsequent allergy/immunology evaluation (13). An etiologic agent for perioperative anaphylaxis may not be identified in 28–50% of patients (Table 1) (3, 14–16).

## Epidemiologic Studies of Penicillin-Induced Perioperative Anaphylaxis

The relatively rare occurrences of true penicillin allergy and perioperative anaphylaxis do collide, but incidence depends on the reporting geographic area (Table 1). Neuromuscular blocking agents have historically been the most common cause of perioperative anaphylaxis analyses in data from mainland Europe, Australia, and New Zealand (17, 18), while beta- lactams are the most commonly identified etiology in the US ad UK (19–22). A US retrospective study examined 717 cases of anaphylaxis, of which 30 were categorized as perioperative anaphylaxis. In this series, antibiotics were the most common cause of perioperative anaphylaxis, with beta-lactams being the most common etiologic agent, and cefazolin as the leading cause (19). A study from the Mayo Clinic reported 30 cases of perioperative anaphylaxis, where antibiotics were found to be the most common cause of an IgE-mediated reaction in nine of 18 patients (21). A prospective study from Massachusetts General Hospital/Brigham identified 25 patients with perioperative anaphylaxis, six of whom reacted to cefazolin. Similarly, in the UK, antibiotics are also the most common cause of perioperative anaphylaxis, with amoxicillin/clavulanate and tecoplanin as the principal etiologic agents (20). Interestingly, the NAP6 allergen exposure survey demonstrated that penicillin allergy impacted the choice of antibiotic in 25% of patients who received vancomycin or teicoplanin, which may contribute to the incidence of anaphylaxis to teicoplanin in the UK (3, 16). The incidence of perioperative anaphylaxis to antibiotics has been rising in France, from 2% in the 1980s to 20% presently, with penicillin and cefazolin as the leading etiologies for anaphylaxis (23). Hepner at al. reported that the incidence of anaphylaxis to penicillin is 1–5 per 10,000 patients, with fatal anaphylaxis occurring in 1–2 per 100,000 treated patients (24).

## Pregnancy, Penicillin, and Peri-partum Anaphylaxis

Pregnancy is an important state to consider the intersection of penicillin allergy and perioperative anaphylaxis. Penicillin G is the preferred agent for Group B *Streptococcus* prophylaxis in pregnancy, and rare cases of anaphylaxis to penicillin-based antibiotics in pregnant women have occurred during labor and delivery (25–30). Most of these patients presented with hypotension that resolved with appropriate treatment, though in one case, there was irreversible fetal neurological damage (31). Hepner et al. highlight cases of anaphylaxis during labor since 2000, with 11 of 13 cases attributable to beta-lactams, five of which were penicillin-based antibiotics (32). A recent systemic review of 12 articles reporting on anaphylaxis during pregnancy found that anaphylaxis in pregnancy occurred during Cesarean section in 49–74% of cases, with beta lactams as the most common cause in 58% of patients, with an allergy work-up only described in two of 12 studies (33). In this analysis, beta-lactam antibiotics became the most common cause of anaphylaxis in the second and third trimesters, whereas first trimester etiologies did not differ from non-pregnant populations (food, venom, drugs) (33).

## Approach to the Patient With a History of Perioperative Anaphylaxis

In most cases of perioperative anaphylaxis, patients have received multiple drugs within a short time frame, therefore, allergy/immunology follow up should be arranged for patients who have experienced perioperative anaphylaxis. Although epidemiologic reports can aid in risk stratification, most patients will need to undergo testing and/or drug challenges in order to undergo future surgical procedures safely. A retrospective review of 73 patients from Massachusetts General Hospital, including 21 patients who reacted to beta lactams, demonstrated that an etiologic agent was identifiable in 18% of patients undergoing allergy/immunology evaluation (34). The number of patients tested to penicillin during part of the study time period was low due to the lack of penicilloyl-polylysine (Pre-Pen, ALK, Round Rock, TX) availability. More importantly, the authors demonstrated that the A/I evaluation allowed 45 of 47 patients to tolerate future anesthesia, with the remaining two patients ultimately being diagnosed with a mast cell disorder (34). Similarly, Gurrieri et al. demonstrated that 23 of 38 patients with a history of perioperative anaphylaxis underwent future surgical procedures at the Mayo Clinic, with those diagnosed with an IgE-mediated allergy avoiding the culprit agent. One patient developed erythema during a subsequent procedure, and 5 patients thought to have non IgE-mediated anaphylaxis were pre-medicated with prednisone and diphenhydramine, and tolerated all anesthetic agents (21). Further United States data from Gonzalez-Estrada and colleagues demonstrated 21 of 30 patients tolerated subsequent surgeries based on allergy evaluation recommendations, with the remaining nine patients not undergoing surgery during the study period (19). A prospective study out of Spain of 473 patients presenting to an outpatient allergy clinic skin prick tested patients to 41 drugs, including antibiotics, trimethoprim–sulfamethoxazole, neuromuscular blocking drugs, latex, iodine, local anesthetics, hypnotics and opioids, regardless of drug exposure history. These patients had a history of an allergic reaction during anesthesia, with 17 reported as having “anaphylactic shock” though this definition was not detailed (35). Through testing, the authors identified that seven of 17 patients with a history of anaphylaxis likely reacted to penicillin. For these and additional non-anaphylactic reactions, there was good correlation with positive penicillin skin testing in cases where penicillin was suspected (35).

## Penicillin and Cephalosporin Cross Reactivity

Another issue that comes up commonly with penicillin in the perioperative period is the cross reactivity between penicillins and cephalosporins. Cefazolin is first line pre-operative antibiotic for most surgical procedures (36, 37), and has been shown to be superior to second line antibiotics, such as clindamycin or vancomycin (38). Cefazolin is also the most common cause of perioperative anaphylaxis in the United States (10, 21, 22, 34, 39). There has traditionally been concern about the use of cephalosporins in patients with a penicillin allergy, but reassuring data regarding the low risk of cross reactivity in patients with a penicillin allergy label are accruing. It should be stated, however, that a true cross reactivity figure is difficult to attain given that the majority of retrospectively studied patients with a reported penicillin allergy are not truly allergic, and penicillin/cephalosporin cross reactivity in patients with confirmed penicillin allergy has not been prospectively studied in large numbers. A retrospective study of 734 surgical procedures in 690 patients, all of whom carried a penicillin allergy label, who received either clindamycin, vancomycin, or cefazolin for preoperative prophylaxis, showed no difference in hypersensitivity reactions regardless of the preoperative antibiotic used, though it is possible that cefazolin could have been avoided in patients reporting higher risk penicillin allergy histories (40). A meta-analysis by Sousa-Pinto and colleagues included 6,147 patients in 77 studies (13 studies in surgical patients exclusively) identified only 44 patients with a cephalosporin allergy who had an index penicillin allergy, calculated as a dual allergy frequency in 0.7% of meta-analysis patients, but the frequency increased to 3% in patients who had a confirmed penicillin allerg (41). In this same study, the frequency of penicillin allergy in patients with reactions to cefazolin was 4.4% in the eight studies looking at this figure (41). It is interesting that patients with an existing cephalosporin allergy have a higher risk of coexisting penicillin allergy. This finding could possibly be explained by the lower incidence of cephalosporin allergy in general (42) as compared to the 10% incidence of reported penicillin allergy. Overall, this retrospective data is reassuring regarding the use of perioperative cephalosporins for most patients reporting an unverified penicillin allergy, but demonstrate a higher risk (3%) in patients with a verified penicillin allergy.

There is also prospective data to support the perioperative use of cephalosporins in patients reporting a penicillin allergy. Sexton et al. sought to implement and evaluate a protocol to increase institutional use of cefazolin in patients with an unverified penicillin allergy label (43). A decision support algorithm was created, and the group examined perioperative antibiotic use in penicillin-allergic patients before and after an educational initiative surrounding the algorithm. They demonstrated an increase of cefazolin use from 34% pre-initiative to >80% (in 756 patients) after the implementation of the algorithm. There were no recorded hypersensitivity reactions and there were no safety reports filed by medical personnel (43). A similar intervention was reported by Grant et al. at Vancouver General Hospital, a tertiary care center in British Columbia, CA. In this intervention, health care team members were educated regarding the policy change of allowing administration of cefazolin in cases of penicillin allergy except with any reactions suggestive of severe cutaneous adverse reactions (SCAR) (44). The authors demonstrated that after the policy change, cefazolin use increased 18%, whereas vancomycin use decreased by over 11%, and clindamycin use decreased by 62%. There were no reports of anaphylaxis or severe allergic reactions in the penicillin allergic patients receiving cefazolin. There were three reports of reactions to cefazolin, but these were in patients who did not carry a penicillin allergy label (44). In summary, these studies strongly support the conclusion that although cefazolin is the most common cause of perioperative anaphylaxis, there does not appear to be a clinically meaningful signal to avoid its appropriate use in patients with an unverified and low risk penicillin allergy history. Consultation with an allergist can be considered in patients with a verified penicillin allergy or high-risk unverified penicillin allergy history.

A barrier to an intervention such as those described by Sexton et al. and Grant et al. above is the clinical decision alert that arises in some electronic health records (EHR) when a cephalosporin is ordered in the setting of any penicillin allergy label. This alert is not in line with current evidence as discussed above, so Macy et al. looked at the effect of removing such a warning from the EHR. Not surprisingly, the authors demonstrated a significant increase in cephalosporin prescribing once the warning was removed, without any concomitant increase in hypersensitivity reactions (45).

## Evaluating Penicillin Allergy Prior to Surgical Procedures

There is neither evidence nor reliable tools to pre-screen patients without a penicillin or cephalosporin allergy prior to surgery for potential allergic reactions to these medications. There is also no evidence to justify widespread screening for mast cell disorders (46). However, in those carrying an unverified penicillin allergy label, a penicillin allergy evaluation prior to a surgical procedure is a way to optimize antibiotic selection and to potentially mitigate perioperative anaphylaxis risk depending on individual historical data. Savic et al. reported their UK experience with direct challenges in patients with a low risk penicillin allergy history. They de-labeled 55 of 56 patients prior to surgery, and of the patients in whom a penicillin-based antibiotic was indicated, 17/19 received the appropriate antibiotic. Patients reported overall reassurance with the process (47). A study from Canada examined the effectiveness of a multi-pronged approach in pre-operative patients. In this study, 194 Patients with a penicillin allergy label were evaluated with penicillin skin testing followed by a challenge if indicated (48). There were four patients with positive skin testing, and 146 of 190 patients underwent oral challenge. The authors demonstrated that the majority (77%) of patients who underwent evaluation received cefazolin, and only five patients received vancomycin. The patients not requiring vancomycin had a mean savings of 22 min of operating room time (48).

Despite the successes reported in the institutional interventions and pre-operative evaluations described above, it is highly likely that further educational work remains. A UK survey including patients and anesthesiologists demonstrated that only 40% of 4,798 responding anesthesiologists would administer penicillin to patients with a low risk reaction history (49). More surprisingly, only 47% would administer penicillin in a patient previously de-labeled by an allergist, due to reported (erroneous) concerns about needing a hospital policy change and/or the inadequacy of a single dose oral challenge if subsequent IV penicillin-based antibiotics were to be used (49). There is admittedly variation in the allergy/immunology literature regarding what constitutes a low risk penicillin allergy reaction history, with general agreement that remote, cutaneous-only reaction histories are low risk, but varied opinions regarding whether hives should be included in the a cutaneous reaction history and what constitutes “remote” (50–52). Further educational efforts will be more effective with a uniform guidance in this area. These efforts should also point out the small, but present increased risk of 3% cross reactivity with cephalosporins in patients with a verified penicillin allergy (41).

## Discussion

Penicillin-based antibiotics and cephalosporins should be used for pre-operative antibiotic coverage as recommended by infectious disease guidelines (36), as the benefit of this coverage outweighs the relatively low risk of perioperative anaphylaxis to these agents. Pregnancy is an area of special consideration both for penicillin allergy and perioperative anaphylaxis. As with surgical prophylaxis, the benefit of using penicillin G for Group B *streptococcus* prophylaxis outweighs the low risk of anaphylaxis. However, in the case that perioperative or peripartum anaphylaxis occurs, penicillins and cephalosporins should be considered as causes of perioperative anaphylaxis given the known statistics reported above. The data support referral for allergy/immunology evaluation after episodes of perioperative anaphylaxis, both to help determine etiologic agent and to make recommendations for future surgical procedures. Optimally, health care systems would automatically refer patients who experience perioperative anaphylaxis to allergy/immunology for this purpose.

**Figure 1:**
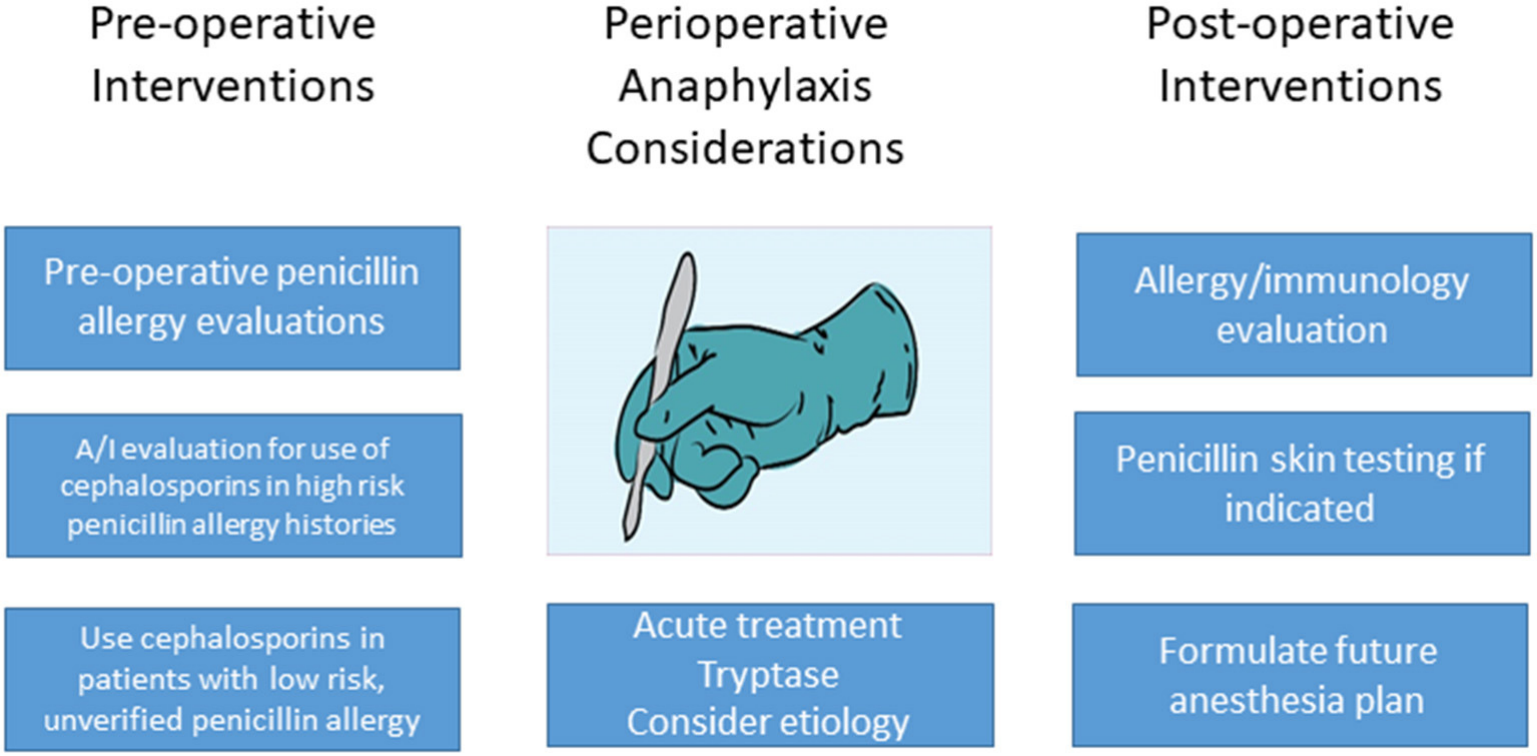
Areas to intervene with penicillin allergy and the perioperative setting.

Although there are well-demonstrated and well-accepted reasons to de-label patients with penicillin allergy (1), the data cited above support the low risk of a hypersensitivity reaction when cefazolin is used for surgical prophylaxis in patients reporting an unverified, low risk penicillin allergy. The data also show that educational efforts are needed to further inform surgical and anesthesia professionals regarding this reassuring data. Such efforts could include academic and health system initiatives, as well as cross specialty collaboration in journals and specialty national meetings. Future interventions should concentrate on optimizing educational and healthcare system approaches among surgical teams to improve antibiotic prophylaxis choice in penicillin allergic patients. Pre-operative penicillin allergy de-labeling is also a feasible, albeit more labor intensive, approach if skin testing is involved. Optimally, graded challenge procedures in low-risk patients would be implemented during pre-operative evaluations with allergy/immunology involvement with such programs. Removing the penicillin/cephalosporin cross reactivity warnings from EHRs in patients with low risk, unverified penicillin allergy labels should be a future goal, given preliminary data regarding the low risk of this occurrence and the known benefit of appropriate antibiotic choice preoperative and otherwise (Figure 1).

For both penicillin allergy and perioperative anaphylaxis, future research should focus on risk stratification and screening tools. Genomic information may aid in predicting those at risk for reacting to penicillin or during surgical procedures. Additional research should be focused on convenient ways to de-label patients prior to surgery, whether through the use of technology (e.g., telemedicine) or employment of challenges at pre-operative appointments. Further outcome data will also help to support removal of the cephalosporin/penicillin cross reactivity warning in the EHR. There is a foundation of data in this area, but such future work will help to optimize pre-operative, operative, and post-operative approach to penicillin-allergic patients.

## Author Contributions

The author confirms being the sole contributor of this work and has approved it for publication.

## Conflict of Interest

The author declares that the research was conducted in the absence of any commercial or financial relationships that could be construed as a potential conflict of interest.

## Publisher's Note

All claims expressed in this article are solely those of the authors and do not necessarily represent those of their affiliated organizations, or those of the publisher, the editors and the reviewers. Any product that may be evaluated in this article, or claim that may be made by its manufacturer, is not guaranteed or endorsed by the publisher.
